# Epigenetic instability caused by absence of CIZ1 drives transformation during quiescence cycles

**DOI:** 10.1186/s12915-023-01671-6

**Published:** 2023-08-15

**Authors:** Olivia G. Dobbs, Rosemary H. C. Wilson, Katherine Newling, Justin F.-X. Ainscough, Dawn Coverley

**Affiliations:** 1 Department of Biology, University of York, York, YO10 5DD UK; 2 York Biomedical Research Institute, University of York, York, UK; 3 Exact Sciences Innovation, The Sherard Building, Oxford Science Park, Edmund Halley Rd, Oxford, OX4 4DQ UK; 4 Genomics and Bioinformatics Laboratory, Bioscience Technology Facility, University of York, York, YO10 5DD UK

**Keywords:** Quiescence, CIZ1, Nuclear condensation, H4K20me1, Epigenetic instability

## Abstract

**Background:**

Cip1-interacting zinc finger protein 1 (CIZ1) forms RNA-dependent protein assemblies that stabilise epigenetic state, notable at the inactive X chromosome in females. CIZ1 has been linked with a range of human cancers and in mice genetic deletion of CIZ1 manifests as hyperproliferative lymphoid lineages in females. This suggests that its role in maintenance of epigenetic stability is linked with disease.

**Results:**

Here, we show that male and female CIZ1-null primary murine fibroblasts have reduced H4K20me1 and that this compromises nuclear condensation on entry to quiescence. Global transcriptional repression remains intact in condensation-deficient CIZ1-null cells; however, a subset of genes linked with chromatin condensation and homology-directed DNA repair are perturbed. Failure to condense is phenotypically mimicked by manipulation of the H4K20me1 methyltransferase, SET8, in WT cells and partially reverted in CIZ1-null cells upon re-expression of CIZ1. Crucially, during exit from quiescence, nuclear decondensation remains active, so that repeated entry and exit cycles give rise to expanded nuclei susceptible to mechanical stress, DNA damage checkpoint activation, and downstream emergence of transformed proliferative colonies.

**Conclusions:**

Our results demonstrate a role for CIZ1 in chromatin condensation on entry to quiescence and explore the consequences of this defect in CIZ1-null cells. Together, the data show that CIZ1’s protection of the epigenome guards against genome instability during quiescence cycles. This identifies loss of CIZ1 as a potentially devastating vulnerability in cells that undergo cycles of quiescence entry and exit.

**Supplementary Information:**

The online version contains supplementary material available at 10.1186/s12915-023-01671-6.

## Background

Quiescence is a reversible, stress-resistant state in which cells experience global transcriptional changes, chromatin condensation, and reduction in nuclear size. In the body, most cells are quiescent and can remain in this stabilised state for years. Some cell types undergo multiple rounds of entry to and exit from quiescence as they execute differentiation programs or adaptive responses. The reversibility of quiescence is essential for maintaining tissue homeostasis and well-studied in the contexts of tissue repair, immune response, and stem cell reactivation, while failure is implicated in tumorigenesis [1]. Cultured mammalian cells can be driven into reversible quiescence by established protocols that impose extrinsic cues to drive signalling, including contact inhibition and nutrient deprivation [2]. Both lead to global gene expression changes and reduce the proliferation rate of cells through repression of cell cycle genes via the dimerization partner, RB-like, E2F, and multi-vulval class B (DREAM) complex [3]. In human fibroblasts, pathway-specific genes have been identified, as well as core quiescence genes, that are both independent of the specific quiescence trigger and vital to maintenance of a long-term quiescent state [4, 5].

Cip1-interacting zinc finger protein 1 (CIZ1) is a nuclear protein implicated in DNA replication [6], cell cycle [7, 8], apoptosis [9, 10], transcriptional regulation [11–13], and maintenance of repressive chromatin at the inactive X chromosome (Xi) [14, 15]. It has been linked with human pathologies including paediatric [16, 17] and common adult-onset cancers [10, 11, 13, 18–20] and with late onset neurological conditions [21] and Alzheimer’s disease [22], often as aberrant alternatively spliced variants [23]. A convincing mechanistic basis for the links with this diverse set of conditions is lacking, and there is no consensus on the underpinning defect or affected pathway. Analysis of the role of CIZ1 at the Xi in differentiated somatic cells identified a functional interaction with the long non-coding RNA (lncRNA) *Xist*, which drives localised formation of CIZ1:RNA assemblies in a manner dependent on CIZ1’s intrinsically disordered regions [24]. Time-resolved high-resolution imaging further suggests that CIZ1:RNA complexes are at the heart of the macromolecular assembly that drives formation of the Xi during early development [25]. The absence of CIZ1 assemblies in differentiated fibroblasts results in failure to enrich histone post-translational modifications (PTMs) that are characteristic of facultative heterochromatin which, along with *Xist* enrichment, can be reinstated upon expression of ectopic CIZ1 [24]. Together, the data argue that CIZ1 assemblies may be part of a phase-separated molecular condensate [26] that promotes or protects a subset of histone PTMs by influencing access to chromatin by modifying enzymes, with possible pleiotropic consequences. Although most of the biochemical evidence around CIZ1 function is derived from analysis at the Xi, its loss is felt at loci across the genome [27], and smaller assemblies are distributed throughout the nucleus in male and female cells [28].

Here, we report the striking observation that loss of CIZ1 combined with quiescence is sufficient to drive formation of proliferative, phenotypically transformed cell lineages. Potential relevance to the earliest, pre-mutation, stages of cancer aetiology focussed our analysis on the immediate response of CIZ1-null cells to the quiescence triggers, rather than the downstream events or resulting lineages. We find that H4K20me1 is depleted in CIZ1-null cells and that this is sufficient to compromise chromatin condensation during quiescence entry and to give rise to a persistent checkpoint activated state from which new cell lineages emerge. Together, the data describe a pathway from epigenetic instability to cellular transformation, initiated by a single gene defect and identifies CIZ1 as a protector of the epigenome relevant to human disease.

## Results

### Nuclear condensation on entry to quiescence is dependent on CIZ1

**Fig. 1:**
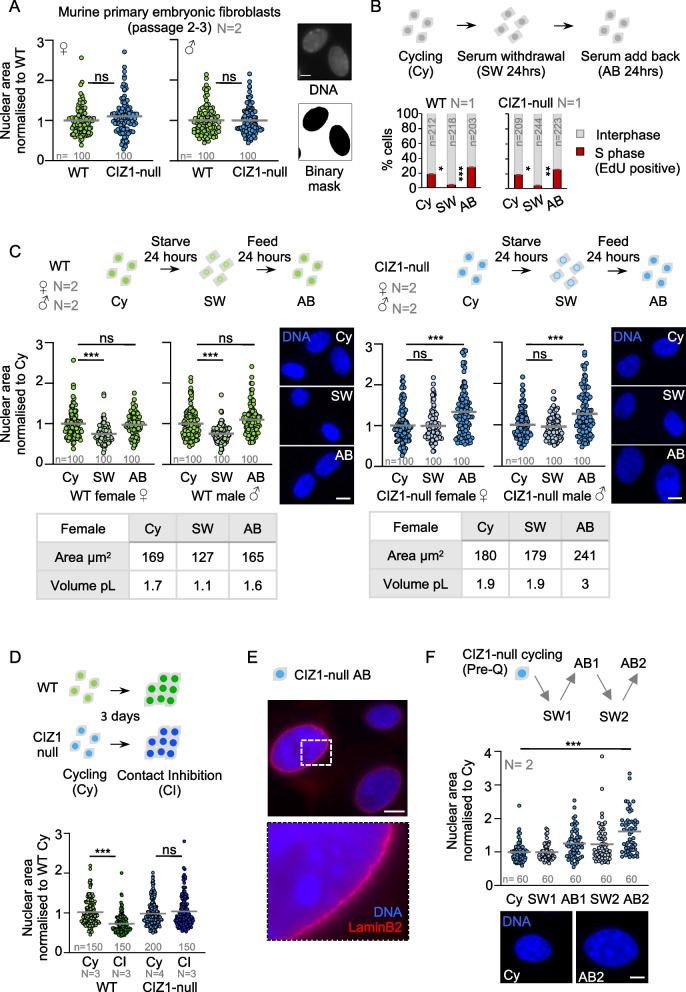
Nuclei from CIZ1-null cells fail to condense on entry to quiescence. **A** Nuclear area in cycling female and male populations of WT (green) and CIZ1-null (blue) primary embryonic fibroblasts (PEFs), demonstrating no difference in nuclear size. Dots represent individual nuclei, with mean (grey bar). Right, example workflow for quantification of nuclear area using Fiji image analysis; see the “Methods” section. **B** Serum withdrawal (SW) and add back (AB) strategy and their effect on S phase index. Histograms show the proportion of cells that incorporate EdU during a 30-min pulse (replicating, red). No difference is detected between WT and CIZ1-null cells. **C** Graphs show change in nuclear area over the SW and AB strategy for male and female WT and CIZ1-null PEFs, normalised to the means for the cycling control state. Representative images of WT and CIZ1-null nuclei at each stage are shown. *Below*, mean areas and calculated volumes assuming a spherical nucleus; see the “Methods” section. **D** Nuclear area for WT and CIZ1-null PEFs in a cycling state (day 0) and at 100% confluency (day 3) with no SW. **E** Immunodetection of the nuclear lamina (Lamin B2, red) in a representative CIZ1-null AB population. **F** Nuclear area for CIZ1-null PEFs over two rounds of SW and AB, showing failure to condense but stepwise decondensation. Representative images demonstrate the overexpansion of CIZ1-null nuclei. All results are either compared by *t*-test (A), one-way ANOVA (B, C, F) or two-way ANOVA (D) where *ns* denotes no significant difference, *p < 0.05, **p < 0.01, ***p < 0.001. DNA is stained with DAPI (blue) and scale bar represents 10 μm

Chromatin condensation and reduction of nuclear size on entry to quiescence is well-documented in *Saccharomyces cerevisiae* [29, 30], primary human fibroblasts [31], and thymocytes [32]. Here, we compared the behaviour of WT murine primary embryonic fibroblasts (PEFs) with that of age-matched PEFs from mice lacking CIZ1 (CIZ1-null) [14]. In a cycling state, no significant difference in average nuclear area was evident (Fig. 1A); however, upon exposure to a trigger of quiescence entry (serum withdrawal, SW) and exit (serum add back, AB), WT and CIZ1-null populations behaved differently. For both, S phase index fell and recovered as expected (Fig. 1B), but only WT cells exhibited a significant 25% decrease in nuclear area relative to parent populations (Fig. 1C). A similar defect was evident in CIZ1-null cells exiting cycle in response to contact inhibition (CI); at 100% confluency, WT nuclear area decreased by 27%, whereas CIZ1-null nuclei did not change (Fig. 1D). Despite this condensation failure, CIZ1-null cells still decondensed their nuclei on cell cycle re-entry (AB), increasing in size by an average of 30% compared to their cycling state (Fig. 1C), while WT nuclei returned to normal size. Considerable heterogeneity was evident in the CIZ1-null population, though even in the most enlarged nuclei DAPI-dense regions remained and the nuclear lamina was unperturbed (Fig. 1E). Similar results were derived from independent primary cell isolates for each genotype (male and female), strongly implicating CIZ1 in the control or execution of nuclear condensation during quiescence. Strikingly, repeated cycles of SW and AB led to progressive nuclear expansion with CIZ1-null nuclei becoming approximately 60% larger than their parent population following 2 rounds (Fig. 1F).

### Global gene expression changes on entry to quiescence are not dependent on CIZ1

**Fig. 2:**
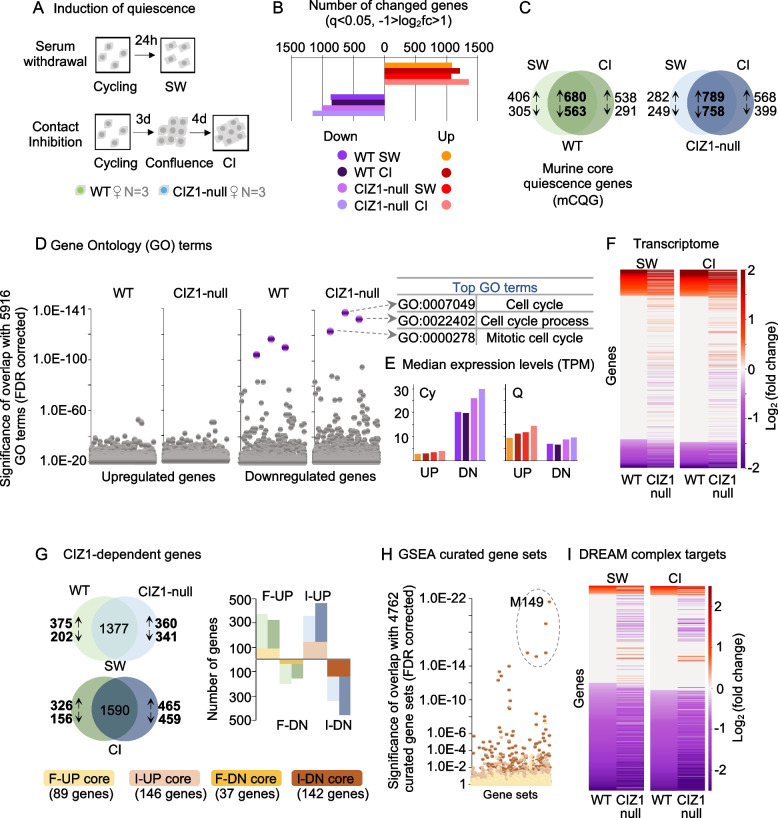
Transcriptome analysis on entry to quiescence. **A** Schematic describing two strategies for inducing quiescence, serum withdrawal (SW, 24 h), and contact inhibition (CI, 7 days). **B** The number of transcription units significantly upregulated (log_2_fc > 1, red) or downregulated (log_2_fc < 1, purple) upon quiescence entry via SW or CI (FDR *q* < 0.05) for WT and CIZ1-null PEFs, showing a similar number in each class. **C** Venn diagrams showing the number of transcription units significantly changed upon entry to quiescence that are common to both quiescence methods, divided into those that go up or down, for WT (green) and CIZ1-null (blue) cells. For WT cells, this defines a murine core quiescence gene set. **D** Dot plots showing the significance of overlap with all 5916 Gene Ontology (GO) terms for the core quiescence gene sets in C. The most significant GO terms (purple) relate to the cell cycle and are returned by the downregulated genes for both genotypes. **E** Median expression level (TPM) of upregulated and downregulated genes shown in B, for cycling and quiescent WT and CIZ1-null cells. **F** Heat maps compare expression (log_2_(fold change)) during quiescence entry between WT and CIZ1-null PEFs, for the two quiescent methods. Transcription units are ordered by WT log_2_(fold change) SW or CI; upregulated (red), downregulated (purple), and where *q* > 0.05 (white). **G** Venn diagrams illustrating CIZ1-dependent genes that are upregulated and downregulated during quiescence entry (intersection shows those that are not CIZ1-dependent). Genes on the left (green) change in WT cells only and are referred to as genes that “fail” (F) to change in CIZ1-null; genes on the right (blue) change in CIZ1-null cells only and are referred to as genes that are “inappropriately” (I) changed in CIZ1-null cells. Right, histogram showing the overlap between the two quiescence methods for CIZ1-dependent genes (F-UP; fail to go up, F-DN; fail to go down, I-UP; inappropriately up, I-DN; inappropriately down), highlighted in shades of brown. **H** Dot plot showing the significance of overlap between the four core CIZ1-dependent gene sets with 4762 GSEA Curated Gene Sets. I-DN (dark brown) overlap most significantly with Fischer DREAM Targets (systematic name M149). The 5 gene sets circled are further analysed in Additional file 6: SDataset 5. **I** Heat maps of all 844 DREAM complex target genes defined in M149 [3], showing expression (log_2_(fold change)) during quiescence entry in WT and CIZ1-null PEFs, for the two quiescent methods. Genes are ordered by WT log_2_(fold change) SW or CI, for both WT and CIZ1-null cells, so that differences in behaviour can be visualised

We asked whether CIZ1 impacts the transcriptional program that normally takes place during quiescence entry [4, 5]. We minimised the effects of pathway-specific signalling and increased focus on common downstream events by comparing changes in the transcriptome in response to two quiescence triggers (SW or CI), in three independently derived populations of PEFs from both WT and CIZ1-null female embryos (Fig. 2A). For WT cells, this generated a gene set that we refer to as murine core quiescence genes (mCQG). This is broadly consistent with the core quiescence program identified previously in human foetal lung fibroblasts [4] (Additional file 1: Fig.S1).

In WT cells, 4.1% and 4.4% of the genome significantly changed expression (-1 > log_2_(fold change) > 1, *q* < 0.05) following SW and CI, respectively. For CIZ1-null cells, a similar response was recorded with 4.4% and 5.3% following SW and CI (Fig. 2B, Additional file 1: Fig.S2A). Full gene lists are given in Additional file 2: SDataset 1 and Additional file 3: SDataset 2.

Integration of quiescence triggers to generate a mCQG for CIZ1-null cells allowed comparison with the WT mCQG (Fig. 2C). WT and CIZ1-null cells returned similar profiles by gene set enrichment analysis (GSEA) [33], and the downregulated component for both gave by far the most significant and coherent overlap with Gene Ontology (GO) terms (Fig. 2D). Unsurprisingly, these relate to the cell cycle, indicating that CIZ1-null cells, like WT cells, remain capable of executing quiescence-linked gene expression programs. This argues that repression is not dependent on nuclear condensation in mammals, which does not align with ideas emerging from analysis in yeast, where condensin-dependent chromatin compaction is a cause of transcriptional repression [30].

Notably, slightly more genes increased than decreased (Fig. 2B) which appears to contradict the widely held notion of global transcriptional repression, though it does support the proposition of quiescence as an actively regulated state [1, 34]. However, when gene expression is expressed in absolute terms (transcripts per million, TPM), it is evident that upregulated genes are typically lower in the cycling state than downregulated genes, which results in greater overall change in the down direction (Fig. 2E). This is consistent with the established view of global transcriptional repression during quiescence.

### A subset of DREAM complex target genes is elevated in CIZ1-null cells

Even though CIZ1-null cells follow the same trend as WT cells, some genes behaved differently (Fig. 2F). These are divided into four categories: those that fail to go up (F-UP) or down (F-DN) in CIZ1-null cells compared to WT cells and those that go up or down inappropriately (I-UP, I-DN) in CIZ1-null cells (because they did not meet the threshold criteria in WT cells, Additional file 4: SDataset 3). Many of these were the same for both quiescence triggers, yielding four high-confidence categories (shades of brown in Fig. 2G). Interrogation of GSEA curated gene sets with the four high-confidence categories (only genes common to both SW and CI) revealed that I-DN genes (brown data points in Fig. 2H) are the most coherent in biological role, with the top set being DREAM complex target genes [3] (Additional file 5: SDataset 4). Analysis of the second (Lee BMP2 targets DN), third (Marson bound by E2F4 unstimulated), fourth (Gobert oligodendrocyte differentiation UP), and fifth (Zhang TLX targets 60 h DN) most significant overlapping gene sets is given in Additional file 1: Fig.S2B and Additional file 6: SDataset 5.

**Fig. 3:**
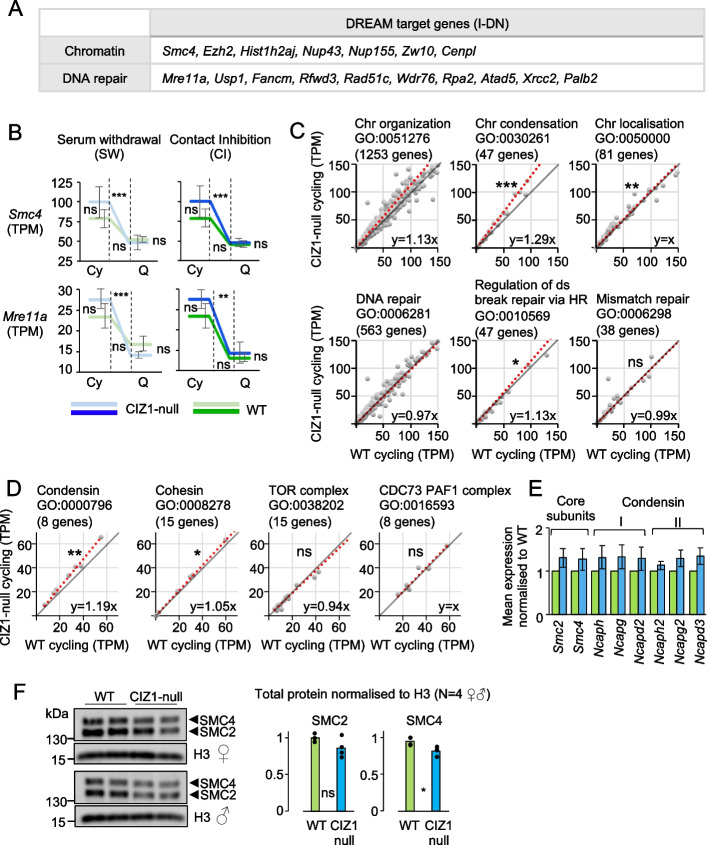
Perturbation of the condensin complex. **A** Genes that relate to chromatin or DNA repair from the 33 I-DN CIZ1-dependent DREAM targets outlined in Additional file 7: Table S1. **B** Expression (mean TPM ± SEM) of *Smc4* and *Mre11a*, before (cycling, Cy) and after (quiescence, Q), by SW (left) and CI (right). Similar data for all 33 I-DN genes is given in Additional file 1: Fig.S3. A gene is defined as significantly changed when the following thresholds are met -1 > log_2_(fold change) > 1 and *q* < 0.05. Significance indicators are ** -1 > log_2_(fold change) > 1 and *q* < 0.01, *** -1 > log_2_(fold change) > 1 and *q* < 0.001, ns denotes no significant difference. **C** Scatter plots comparing expression (mean TPM) in cycling WT and CIZ1-null cells, for all genes within high level GO terms chromosome organisation and DNA repair, plus two subordinate terms for each, as indicated. The grey line (*y* = *x*) illustrates no difference between WT and CIZ1-null cells and the red line is the best fit to the data calculated in excel, with the slope of the line indicated. A skew towards the *y* axis indicates a higher expression in CIZ1-null cycling cells. **D** As in **C**, for subunits of the condensin complex, the cohesin complex, the TOR complex, and the CDC73 PAF1 complex. Expression of the condensin subunit class is significantly affected by loss of CIZ1. **E** Mean TPM for all condensin subunits in WT (green) and CIZ1-null (blue) PEFs in the cycling state, normalised to WT, ± SEM. *Smc2* and *Smc4* are the two core subunits common to both condensin I and II. Individually, no subunit is significantly different between WT and CIZ1-null. **F** Whole cell lysates from WT and CIZ1-null cycling populations, female (top) and male (bottom), illustrating the total protein levels for SMC2, SMC4, and histone H3. Right, quantification showing mean for the 4 independent populations (2 female, 2 male) with individual values indicated. Results are compared by *t*-test (F) or the Wilcoxon’s signed ranked test (C, D) where *ns* denotes no significant difference, *p < 0.05, **p < 0.01, ***p < 0.001

The DREAM complex is best known for its role in quiescence where it is involved in repression of pro-proliferation genes. Looking at the 929 genes normally under the regulation of the DREAM complex (GSEA M149), we saw differences in the behaviour of a subset during quiescence entry (Fig. 2I) with 33 of them represented in our I-DN set (listed in Additional file 6: SDataset 5). When classified by biological process, the most focussed GO terms returned by these high-confidence CIZ1-dependent I-DN DREAM target genes relate to DNA metabolism (repair, recombination, replication, and chromatin) (Fig. 3A, Additional file 7: Table S1). Similarly, the GO terms returned by the I-DN genes that are part of the next four most significant overlapping GSEA curated gene sets also relate to the same processes, most notably chromatin and response to DNA damage (Additional file 6: SDataset 5).

Investigation of the TPM values for the 33 DREAM target genes showed that, in WT and CIZ1-null cells, all genes are downregulated on entry to quiescence. However, the fold change does not reach the significance threshold (-1 > log_2_(fold change) > 1 and *q* < 0.05) in WT cells. TPM values start higher (cycling state) and in many cases fall further (quiescent state) in CIZ1-null cells compared to WT. The additive effects of these differences contribute to the genes only being significant in CIZ1-null (Fig. 3B and Additional file 1: Fig.S3). From this, we can see that elevated expression in the cycling state appears to be a major contributory factor to their emergence through our filters. This led us to explore the cycling state, focussing on the GO terms that were most represented.

The high-level term “chromosome organisation” slightly favours higher expression in CIZ1-null cells, and this trend is even more marked for the subordinate term “chromosome condensation” but not for “chromosome localisation.” Similarly, “regulation of DNA double-strand break repair by homologous recombination” is slightly skewed while the similar size term “mismatch repair” and their parent term “DNA repair” are not (Fig. 3C). This suggests that chromatin condensation and homology directed repair are both affected by loss of CIZ1, offering candidate processes underpinning the links between CIZ1 and disease. Here, we have focused on chromatin condensation because of the clarity of the result, and the observed phenotype of CIZ1-null cells (Fig. 1).

In eukaryotes, the condensin complex exists in two forms, I and II. Both contain the core subunits SMC2 and SMC4, of which SMC4 is represented in our core I-DN set. Condensins have been associated with chromatin compaction during yeast and human quiescence [4, 30], and condensin II specifically implicated in a thymocyte model of quiescence [32]. Strikingly, expression of *Smc2*, *Smc4* and the additional subunits *Ncaph*, *Ncapd2*, and *Ncapg* for condensin I, and *Ncaph2*, *Ncapd3*, and *Ncapg2* for condensin II are all skewed towards increased expression in CIZ1-null cycling cells compared to WT. Though the difference is not significant for any one component, the overall trend is significant (Fig. 3D, E). In contrast, subunits of the closely related cohesin complex are less affected. Additionally, two similarly sized sets that represent the TOR complex involved in cell proliferation control [35], and the CDC73 PAF1 complex involved in survival during long-term quiescence [36], are not affected (Fig. 3D). Thus, the observed deficit in nuclear condensation during entry to quiescence (Fig. 1) correlates with elevated expression of the condensation machinery prior to receipt of quiescence triggers. SMC2 and SMC4 total protein levels were relatively unchanged (Fig. 3F); therefore, elevated transcript is more likely to reflect compensation for compromised function.

### Reduced H4K20me1 in CIZ1-null cells

Function of the condensin II complex has been linked to the methylation status of histone H4 lysine 20 (H4K20). Normally, dissociation of the demethylase PHF8 leads to emergence of H4K20me1 at mitosis and recruitment of NCAPD3 and NCAPG2 [37], while lack of the mono-methyltransferase SET8 results in mitotic chromosome condensation failure [38]. In addition to mitosis, H4K20 methylation has been implicated in quiescence-linked condensation through loss of the methyltransferase KMT5C/Suv4-20h2 [31].

**Fig. 4:**
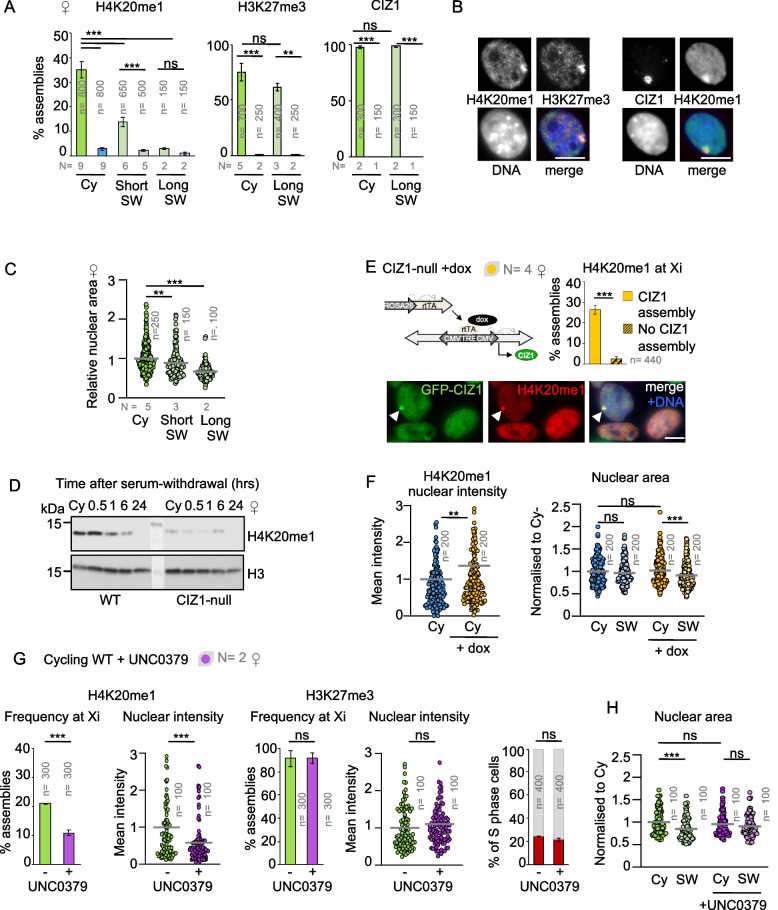
H4K20me1 depletion in CIZ1-null PEFs. **A** Frequency of H4K20me1, H3K27me3, and CIZ1 assemblies at the Xi in female WT (green) and CIZ1-null (blue) PEFs, in the cycling state (darker shades) and after a short or long SW (paler shades), with ± SEM. *n* denotes the total number of cells evaluated. **B** Example images of H4K20me1 (green) at H3K27me3-marked (red) Xi and H4K20me1 at CIZ1-marked (red) Xi in WT cells. **C** Nuclear area for female WT PEFs demonstrating a gradual drop in size over a short and long SW window. **D** Western blot illustrating H4K20me1 levels over a 24-h SW time course for WT and CIZ1-null female PEFs and histone H3. **E** Strategy describing GFP-CIZ1 doxycycline (dox) induction in CIZ1-null PEFs [14]. Right, effect on frequency of H4K20me1 assemblies in a dox-induced CIZ1-null female cycling population (yellow) after 48 h, categorised by whether GFP-CIZ1 expressing cells had formed a distinct CIZ1 assembly. Below, representative image illustrating colocalisation of GFP-CIZ1 (green) and H4K20me1(red) assemblies (white arrow). **F** Mean H4K20me1 immunofluorescence signal in nuclei from CIZ1-null cycling PEFs with (yellow) or without (blue) induction of GFP-CIZ1 (+ dox). Right, nuclear area in the same cells following a long SW, showing restoration of condensation capability. **G** WT cycling PEFs before (green) and after a 2-h incubation with 10 μM UNC0379 (purple), showing reduced H4K20me1 assemblies (histogram) and fluorescence intensity (dot plots), but no effect on H3K27me3. Right, histogram shows no effect on the proportion of replicating cells (red), detected after 30 min of EdU labelling. **H** Effect of UNC0379 on nuclear area before and after a short SW, showing loss of function in female WT PEFs. Results are compared by *t*-test (E, F (left), G), one-way ANOVA (C), or two-way ANOVA (A, B, F (right), H) where *ns* denotes no significant difference, *p < 0.05, **p < 0.01, ***p < 0.001. DNA is stained with DAPI (blue) and scale bars represent 10 μm

Our previous analysis of CIZ1 at the Xi revealed CIZ1-dependent maintenance of H3K27me3 and H2AK119Ub1 [27]. As H4K20me1 is also known to be enriched at the Xi and suggested to play a role in its compaction (though not its repression) [39], we evaluated H4K20me1 in CIZ1-null cells (Fig. 4A). This showed a dramatic reduction in frequency of H4K20me1-enriched Xi’s in female CIZ1-null cycling populations (3%) compared to WT (35%) and aligns with results for both CIZ1 itself and H3K27me3 (Fig. 4A, B) and with previous analysis of H2AK119Ub1 [27]. Notably different is the behaviour of H4K20me1 upon SW. While CIZ1 and H3K27me3 remained high in WT cells, the frequency of H4K20me1-enriched Xi’s fell to 14% after 1 h (short SW) and 3% by 24 h (long SW). Therefore, H4K20me1 enrichment at the Xi is normally modulated during entry to quiescence (Fig. 4A), coincident with nuclear condensation (Fig. 4C). Both SW-induced loss of H4K20me1 in WT cells and overall suppression in CIZ1-null cells are evident globally in western blot analysis. This is marked in female cells (Fig. 4D) but moderate and later in male cells which do not contain Xi-associated enriched zones (Additional file 1: Fig.S4A). H4K20me1 depletion in CIZ1-null cells (cycling and SW) was further confirmed in male cells by immunofluorescence (Additional file 1: Fig. S4B). Depletion of both Xi-enriched and nucleus-wide H4K20me1 confirms that the establishment or maintenance of H4K20me1 is dependent on CIZ1 and that this defect exists before exposure to quiescence stimuli.

To confirm the link between CIZ1 assemblies (the RNA-dependent concentration of CIZ1 observed around the Xi) and accumulation of H4K20me1 at the Xi, we rescued the phenotype in CIZ1-null cells by induced expression of ectopic GFP-CIZ1 from an integrated vector (Fig. 4E) [14]. All nuclei express GFP-CIZ1, but only a subset form new discrete assemblies within 48 h (18% in the population analysed here). For these cells, 26% show enrichment of H4K20me1, compared to only 2% in nuclei with no CIZ1 assemblies (Fig. 4E). This aligns with previous results which showed coincident accumulation of H3K27me3, H2AK119Ub1, and *Xist* with new CIZ1 assemblies [27]. Ectopic GFP-CIZ1 also increased the mean nuclear intensity of H4K20me1, and importantly supported partial reversion of the condensation defect after SW (Fig. 4F), reinforcing the link between CIZ1 and condensation during entry to quiescence.

### Inhibition of SET8 is sufficient to compromise nuclear condensation in quiescence

CIZ1 stabilises multiple PTMs which have the potential to cause pleiotropic effects. Therefore, we tested whether depletion of H4K20 methylation alone can mimic the CIZ1-null condensation defect in WT cells. The substrate competitive inhibitor UNC0379 selectively inhibits SET8 [40] and was effective at reducing the frequency of H4K20me1-enriched Xi’s and nucleus-wide H4K20me1 levels in WT PEFs during a 2 h incubation, with no effect on H3K27me3, S phase index (Fig. 4G), or nuclear size in cycling populations (Fig. 4H, Additional file 1: Fig. S4C). However, following a short SW, condensation was impaired in UNC0379-treated WT nuclei which phenocopies the effect of deleting CIZ1 (Fig. 4H, Additional file 1: Fig. S4C). This implicates H4K20me1 depletion as the defect underlying failure to condense but does not rule out the possibility that loss of CIZ1 might mediate its effect in a different way.

### A pathway from epigenetic instability to spontaneous transformation

**Fig. 5:**
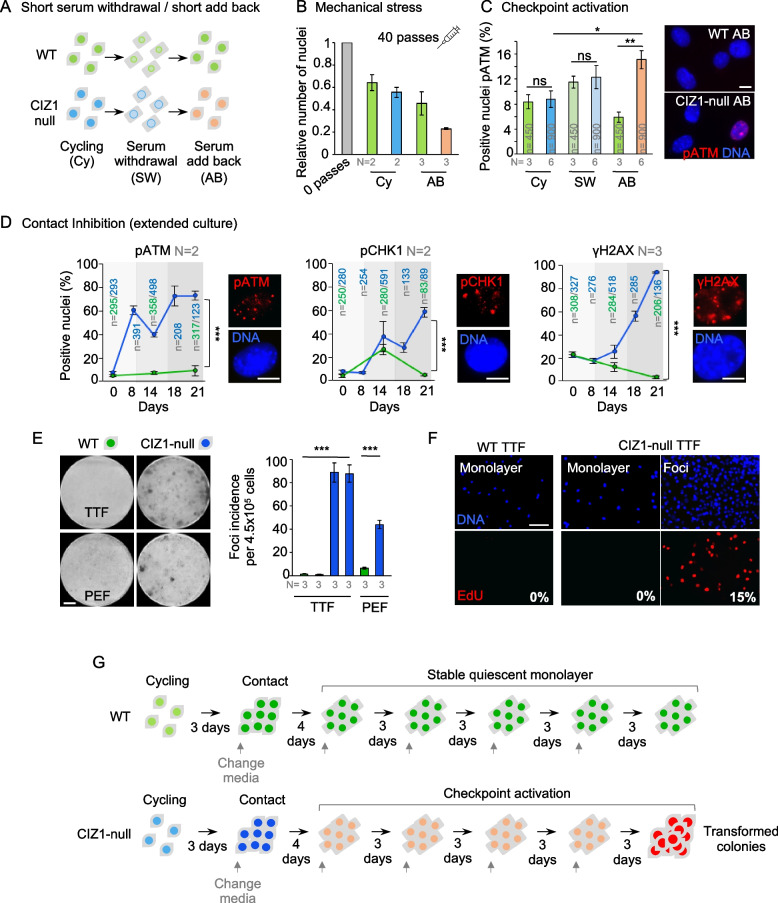
Fragility, checkpoint activation, and instability induced by quiescence in CIZ1-null cells. **A** Short SW and AB strategy used to analyse the response of WT (green) and CIZ1-null (blue) nuclei in B and C. **B** The relative number of nuclei that survived 40 passes through a 25G needle [41] ± SEM, where *N* represents the number of independent experiments, each with 2–7 fields analysed. **C** Frequency of cells with activated checkpoint kinase (pATM), ± SEM. Right, representative images showing pATM foci (red) in WT and CIZ1-null AB nuclei. **D** Checkpoint activation (pATM, pCHK1, yH2AX) in WT and CIZ1-null cells after CI and extended culture, where day 0 represents cycling cells. Below, representative images show foci in contact inhibited CIZ1-null cells at the end of the 21-day time course. **E** 6 cm culture dishes stained with crystal violet showing primary fibroblasts (tail-tip, TTF and embryonic, PEF) populations from WT and CIZ1-null mice, at the end of the 21-day CI time course. Bar is 1 cm. Right, quantification of macroscopically visible foci in CIZ1-null TTF populations and a PEF population, compared to WT. Histograms show mean foci incidence ± SD at 21 days after plating 4.5 × 10^5^ cells. *N* represents the number of plates counted. **F** Field images of WT and CIZ1-null monolayers 21 days post-plating, detected after a 16-h pulse with EdU. Monolayer cells remain unlabelled however CIZ1-null focal outgrowths contain S phase cells. Scale bar 200 μm. **G** Schematic representation of the order of events observed in CIZ1-null cells following CI and extended culture. Results are compared by *t*-test (D, E) or two-way ANOVA (C) where *ns* denotes no significant difference, *p < 0.05, **p < 0.01, ***p < 0.001. DNA is stained with DAPI (blue), and scale bar represents 10 μm unless otherwise stated

To explore the impact of defective H4K20me1-dependent condensation and inappropriate decondensation, we compared the properties of nuclei at SW and AB. Susceptibility to shear forces induced by passage through a fine needle [41] was increased in CIZ1-null nuclei upon AB (Fig. 5A, B), correlating nuclear expansion with nuclear fragility. This suggests that CIZ1-null cells undergoing quiescence cycles in the body might be vulnerable to mechanical stress. However, even without mechanical stress, increased phosphorylation of ataxia telangiectasia mutated (pATM) indicates that cell cycle checkpoints are significantly more activated in CIZ1-null PEFs specifically upon AB (Fig. 5C) and so raises the possibility of DNA damage. A similar response was seen following CI using a standardised long-term maintenance protocol in which media is replenished every 3 to 4 days (natural SW by depletion). Under these conditions, checkpoint activation (pATM) was evident within the first week, while phosphorylated checkpoint kinase 1 (pCHK1) and phosphorylated histone H2AX (γH2AX) emerged later. For all markers, there was no difference between the two genotypes at the start of the time course (cycling), but all became progressively activated, reaching 60–90% of the CIZ1-null population (Fig. 5D). This suggests that CIZ1-null cells are either impaired in their ability to resolve DNA damage or associated signalling, or that they experience repeated assault.

By the end of the 21-day CI time course, proliferating colonies emerged from checkpoint activated CIZ1-null monolayers (at a rate of approximately 1 per 5000–10,000 cells plated), while WT cells established a stable monolayer (Fig. 5E). This was replicated in independent isolates of both PEFs and dermal fibroblasts from tail tips (TTFs). Cell proliferation is evidenced by incorporation of the nucleotide analogue EdU into approximately 15% of colony cells, but not the surrounding monolayer (Fig. 5F). Checkpoint activation (γH2AX) remained evident, and colony cells exhibited features characteristic of transformed cells, encroaching on surrounding monolayers (Additional file 1: Fig. S4D, E). A spontaneously immortalised TTF line also presented with substantially enlarged nuclei compared to parent cells (Additional file 1: Fig. S4F). These data show that the epigenetic vulnerability caused by CIZ1 deletion is sufficient to create vulnerable nuclei, and to drive quiescence escape (Fig. 5G).

## Discussion

We have identified a requirement for CIZ1 during the formation of a quiescent nucleus and outlined a potentially catastrophic series of events that take place in its absence. Previous analysis showed that CIZ1-*Xist* assemblies modulate H3K27me3 and H2AK119Ub1 at the Xi [24, 27], extended here to include H4K20me1. Biochemical studies with purified components revealed two separate RNA interaction interfaces in CIZ1 [24], and deletion studies showed a functional relationship with the repeat E element of *Xist* [14, 15]. Multivalent interaction with RNA (and protein self-interaction) has the potential to generate a network or “matrix” [24], and *Xist* has the capability to specify where such matrices will form. Moreover, by trapping and amplifying local concentrations of *Xist*, CIZ1 appears to augment the action of other *Xist*-dependent activities such as those that modify histones. Thus, CIZ1 is emerging as a protector of the epigenetic landscape, with likely pleiotropic and context-specific effects. One of these, nuclear condensation failure, is the consequence of H4K20me1 depletion and manifests during quiescence entry.

However, the data suggest that it is subsequent inappropriate decondensation on re-entry to cycle that is most damaging as it is accompanied by nuclear fragility and sustained activation of the DNA damage response. Crucially, this precedes the emergence of phenotypically transformed lineages, suggesting that the epigenetic instability experienced in the absence of CIZ1 could be a driver of genome instability and possibly therefore an early (pre-mutation) driver of cancer initiation. In fact, aberrant nuclear architecture is a long-established feature of cancer cells, with diagnosis of some cancers historically based on increases in nuclear size or the condensation state of chromatin [42]. This implicates condensin function, though it is also linked with changes in ploidy. In our protocols, oversized nuclei can be generated in short-term experiments, with cycles of SW and AB that fall far short of a replication cycle (1 h) and are therefore not primarily driven by changes in ploidy. Thus, we propose that nuclear expansion may be an early event that precedes genetic and ploidy change in some cancers.

Aberrant condensin expression and mutations in condensin II have been reported in colorectal and pancreatic cancers [43–45] and linked with chromosome instability and disease progression. In murine models, mutations in condensin II subunits cause defects in T cell development, failure to condense, and development of T cell lymphoma [32, 46]. This parallels the abnormal quiescence of CIZ1-null fibroblasts in this study and the pathology of CIZ1-null mice. Leukaemia [47] and high-penetrance lymphomas [14] are evident in adult mice bearing exon 5 deletion or gene trap insertion into intron 1, respectively. Notably, both cell types originate in progenitors whose maintenance requires quiescence entry and exit cycles [48]. This aligns with the idea that a defect in CIZ1 could underpin the emergence of unstable lineages in cell types that undergo quiescence cycles as part of their normal biology.

The balance between histone modifiers that add and remove PTMs and determine the methylation dynamics of H4K20me1 are also implicated in cancer: SET8 with hepatocellular carcinoma [49] and PHF8 with colorectal cancer [50]. Therefore, their inhibition may offer a route to intervention. This idea is supported by high-frequency point mutations in histone genes in several tumours, notably paediatric cancers [51, 52]. However, before exploitation of such findings, it will be necessary to understand the downstream cellular events that are compromised, and crucially when in the aetiology of cancer, they give rise to irreversible genetic change. Here, we have uncovered one such consequence and identified quiescence entry and exit as a vulnerable cellular transition.

Our data does not show that CIZ1-null cells experience DNA damage prior to phenotypic transformation. However, it does show persistent activation of the checkpoint kinases that arrest the cell cycle in response to DNA damage. In pre-malignancies, chronic checkpoint activation is a well-established concept that offers a selection pressure in favour of checkpoint escape and the subsequent emergence of unprotected hypermutable cells [53, 54]. Thus, quiescence cycles are emerging as a point of vulnerability and possible intrinsic driver of transformation.

Understanding the immediate trigger of checkpoint activation is not straightforward. It could be a direct response to aberrant condensation [55] possibly detected at decondensation. However, it seems more likely that it is initiated by DNA damage resulting from hyper-decondensation. We also cannot rule out other CIZ1-dependent chromatin deficiencies that directly compromise DNA repair. CIZ1-null cycling cells are sensitive to replication stress induced by hydroxyurea, suggesting a defect in the resolution of DNA breaks unconnected to quiescence [47]; however, these CIZ1-null cells could have already experienced quiescence cycles and developed susceptible nuclei. Similarly, a separate study reported sensitivity to γ-irradiation in fibroblasts and increased DNA breaks in brain tissue, linking the deficit to impaired motor and cognitive functioning in mice [21]. Here again, the source of the DNA breaks is not known and could have arisen from a condensation defect. Finally, our transcriptomic study detected elevated expression of regulators and executors of homology-directed DNA double-strand break repair, which could reflect either compromised functionality, or heavy burden of damage.

## Conclusions

Our findings demonstrate that CIZ1 is required for maintenance of H4K20me1 and for H4K20me1-dependent condensation, which protects against aberrant nuclear expansion and instability during quiescence cycles. Whether compromised CIZ1 assemblies and the associated epigenetic instability is ever a primary cause for transformation outside of genetically manipulated murine models remains to be seen. However, the data described here outline a pathway which, through aberrant quiescence cycles, translates epigenetic instability to cellular transformation and so identifies CIZ1 as a guardian of the epigenome with implications for human disease.

## Methods

### Cells and cell culture

CIZ1-null mice were generated as described [14] from C57BL/6 ES clone IST13830B6 (TIGM) harbouring a neomycin resistance gene trap inserted into intron 1. Murine primary embryonic fibroblasts (PEFs) were derived from individual embryos at days 13–14 of gestation and murine tail-tip fibroblasts (TTFs) at 3–4 weeks (Additional file 7: Table S2). Both were maintained in Dulbecco's Modified Eagle Medium (DMEM), GlutaMAX, high glucose (4.5 g/l), and low glucose (1 g/l) (Gibco), respectively, and grown on Nunc™ Cell Culture Dishes (ThermoFisher Scientific, 150,350) with 0.13–0.16 mm thick glass coverslips. Media was supplemented with 10% FBS (PAA) and 1% Pen/Strep/Glutamine (PSG, Gibco) referred to here as high-serum media. Cells were maintained in a rapidly cycling state at 37 °C with 5% CO_2_ and split to avoid cell contact. All cell populations were used at passages 2–4. For inducible cells harbouring transactivator and responder transgenes, addition of doxycycline (dox) to media (5–10 μg/ml) was used to induce GFP-CIZ1 over 48 h.

### Quiescence

For quiescence by serum withdrawal, cells were plated at 70–80% confluency in high-serum media. After 24 h, media was removed, cells were washed in warmed PBS (Dulbecco’s Phosphate Buffered Saline, 14,190–094), and low-serum media added (DMEM, 0.01% FBS and 1% PSG) [2] for either a short (up to 2 h) or a long (24 h) serum withdrawal. To achieve contact inhibition, fibroblasts were plated at 70–80% confluency and cultured to 100% confluency (typically 3 days). At the point of 100% confluency, media was changed (fresh, high-serum media), and cells were incubated for a further 4 days (quiescent). For foci formation, quiescent cells were maintained for a further 2 weeks with high-serum media changed twice a week.

### Detection of foci formation

Plates with adherent cells were washed twice in room temperature (RT) PBS, followed by fixation with 4% paraformaldehyde (PFA) for 20 min, then stained with 0.05% filtered crystal violet for 30 min, and washed to reveal stained foci. Analysis was in triplicate, imaged using bright-field microscopy, and foci scored manually and expressed as a function of cells plated.

### Inhibitors

UNC0379 (Sigma, sml1465), a selective, substrate competitive inhibitor of SET8 (IC_50_ of 7.3 μM [40]), was used at 10 μM for 2 h (in high-serum media for cycling cells or low-serum media for cells subject to serum withdrawal).

### S phase labelling

Adherent cells on coverslips were incubated with 5-ethynyl-2′-deoxyuridine (EdU, 10 μM) for a 30-min pulse period, or extended time periods where indicated, under standard growth conditions. To visualise newly synthesised DNA, coverslips were washed briefly in cytoskeletal buffer (CSK; 10 mM PIPES/KOH pH 6.8, 100 mM NaCl, 300 mM sucrose, 1 mM EGTA, 1 mM MgCl_2_, 1 mM DTT, 1 cOmplete™ Protease Inhibitor Cocktail per 50 ml) with 0.1% Triton-X-100 before fixation with 4% paraformaldehyde (PFA) for 15 min. Coverslips were then washed in PBS and EdU detected using the Click-iT® EdU Alexa Fluor® 555 kit (ThermoFisher), as recommended. Briefly, coverslips were blocked with BSA Antibody buffer (0.02% SDS, 0.1% TX100, 10 mg/ml nuclease-free BSA in PBS) before incubation in a light-proof humidified chamber with Click-iT® reaction cocktail for 60 min. Coverslips were then washed and mounted using VectaShield with DAPI (Vector Labs).

### Immunofluorescence

Adherent cells grown on glass coverslips were subjected to a 1 min detergent wash (0.1% Triton-X-100 in PBS) and then fixed for 15 min in 4% PFA. After fixation, cells were rinsed twice with RT PBS and then incubated for 30 min in BSA antibody buffer. Coverslips were incubated for 1–2 h at 37 °C with primary antibody (in BSA antibody buffer), followed by three washes in BSA antibody buffer before incubating with anti-species secondary antibodies (Alexa Fluor 488 or 568) for 1 h. Cells were washed a further three times in BSA antibody buffer prior to mounting in VectaShield with DAPI (Vector labs, H-1200). For DNA damage response antibodies (pATM, pH2AX, pChk1), PBS was substituted by TBS in all buffers. Antibodies are listed in Additional file 7: Table S3.

### Imaging and image analysis

Fluorescence was captured using a Zeiss Axiovert 200 M fitted with a 63X/1.40 Plan-Apochromat objective and Zeiss filter sets 2, 10, 15 (G365 FT395 LP420, BP450-490 FT510 BP515-565, BP546/12 FT580 LP590), using the Axiocam 506 mono and Axiovision image acquisition software (SE64 release 4.9.1). For images where intensity differences are quantified, all samples were analysed as a set, with constant image acquisition parameters, and no image manipulation. For presentation purposes, images were enhanced using Fiji V1.0 [56], and where intensity differences are being illustrated, all images in the set were manipulated identically. All quantification was conducted on unedited images.

For measurements of nuclear area, typically 30–50 cells from each population were analysed using Fiji V1.0 [56]. Channels were split to show blue, green, and red output separately, and thresholds for nuclear area are identified using the Otsu setting in the blue (DAPI) channel. Thresholds were converted to masks, and all particles within size 0.01-Infinity were analysed and returned as area in pixels. Conversion to area in μm^2^ then to volume was calculated based on the 10 μm scale bar and assumes a spherical nucleus. Where intensity measures for other channels were required, the DAPI mask was overlaid onto the relevant image and pixel density within the mask returned as maximum, minimum, or mean density in the selected area.

### Western blots

Adherent cells were scrape harvested following two cold washes in PBS supplemented with 2 mM PMSF and then either denatured by heating to 90 °C in SDS-PAGE sample buffer (2% SDS, 15% glycerol, 1.7% betamercaptoethanol, 75 mM Tris pH 6.8 with bromophenol blue) to generate whole cell lysate or supplemented to 0.1% Triton-X-100. After Triton-X-100 incubation for 3–5 min on ice, samples were centrifuged for 2 min at 5000 rpm, to generate a pellet fraction (insoluble material including chromatin). Fractions were denatured in SDS-PAGE sample buffer for subsequent analysis by western blot after separation through a 4–15% gradient gel (BioRad) and transfer to nitrocellulose membrane using iBlot gel transfer stacks (Invitrogen) or a semi-dry transfer. Membranes were blocked with 5% BSA or 10% milk, in PBS with 0.1% Tween20, for 30 min before primary antibody incubation for 2 h at RT or overnight at 4 °C depending on antibody, with gentle agitation. Blots were washed three times with blocking buffer then probed with HRP-conjugated anti-species secondary antibody (Jackson Immunochemicals 115–035-174 and 211–032-171) for 1 h at RT and imaged using EZ-ECL (Biological Industries) and Syngene PXi chemiluminescence imaging system. Image quantification was performed on unedited images (Additional file 8) with background subtracted and normalised to histone for loading.

### Nuclear fragility

Method was adapted from Furusawa, T. et al. [41]. Adherent cells were harvested by trypsinisation then pelleted at 2000 rpm and resuspended in 200 μl PBS/Hoechst (1:1000). Before subjecting cells to mechanical shear, a reference sample (0 passes) was taken and fixed in 4% PFA to generate a measure of cell count and integrity. Using a 1ml syringe, the remaining cell suspension was passed through a 25G needle 40 times and then fixed. Equal volumes of treated and untreated fixed cells were concentrated onto microscope slides using a cytospin and mounted in Vectashield (Vector Labs, H-1000). The number of intact stained nuclei in 2–7 fields per independent experiment were counted, averaged, and expressed relative to control.

### Graphical presentation and statistical analysis

Experiments were designed to use the minimum number of animals (independently derived PEF or TTF populations) while achieving statistically valid data, with *N* representing the number of independent experiments. Unless otherwise stated for nuclear area measurements, 30 or 50 cells from three independent coverslips were analysed at the indicated treatment times. For scoring histone PTMs, or protein at the Xi, each replicate is an average of 3 independent counts or measurements per coverslip (30–50 cells each), with the average of all replicates shown. Unless otherwise stated, values represent the means ± SEM. Asterisks indicate statistical significance (ns not significant, ∗ *p* < 0.05; ∗∗ *p* < 0.01; ∗∗∗ *p* < 0.001). Statistical analysis was carried out in GraphPad Prism using a two-tailed Student’s *t* test, one-way ANOVA, or two-way ANOVA. Significance for expression level differences between WT and CIZ1-null cycling cells for the GO terms chromosome condensation, chromosome localisation, regulation of DNA double-strand break repair by homologous recombination, mismatch repair and the subunits of the condensin complex, cohesin complex, TOR complex, and CDC73 PAF1 complex were calculated using the Wilcoxon signed rank test. Graphs were generated using GraphPad Prism Version 9.1.0 (216) or Microsoft Excel and, where indicated, measurements were normalised to the relevant internal control.

### Transcriptome analysis

Primary (before passage 4) murine embryonic fibroblasts 13.1, 13.8, and 14.4 (female WT PEFs) and 13.15, 13.17, and 14.2 (female CIZ1-null PEFs) were grown to 80% confluency. For each cell line, RNA was extracted with TRIzol (Ambion, 15,596–026), from cycling cells, 24 h following serum withdrawal (SW), or 4 days after 100% confluency (CI). Briefly, adherent cells were washed twice with PBS, drained on a shallow angle for 2 min and excess PBS removed. One millilitre of TRIzol was added per 28 cm^2^ and incubated for 3–5 min at RT with periodic agitation. Lysates were collected in clean Eppendorf tubes. Chloroform was added to lysates at a ratio of 1:5, and lysates were shaken vigorously for 15 s before incubation at RT for 3 min before centrifugation at 12,000 g for 15 min. The aqueous phase was transferred into a clean Eppendorf and mixed with equal volume of isopropanol through gentle inversion and incubated at RT for 10 min followed by a 10 min centrifugation at 12,000 g. Supernatant was removed, and the RNA pellet washed with an equal volume of 75% ethanol to the volume of TRIzol used. Sample was centrifuged for 5 min at 12,000 g and the supernatant removed. RNA pellet was allowed to dry for 30 s before resuspension in nuclease-free water. Isolated RNA was then treated with DNase (Roche, 04716728001) and purified (RNA Clean & Concentrator-5 kit, R1015, Zymo Research), before quality analysis by agarose gel, NanoDrop spectrophotometer, and Agilent 2100 Bioanalyzer. Libraries were prepared with NEBNext® Ultra™ RNA library Prep Kit for Illumina® and enriched for mRNA using NEBNext Poly(A) mRNA Magnetic Isolation Module, which is optimised for production of libraries with 250–400 bp inserts. Enriched mRNA was fragmented by heating to 95 °C for 12 min, cDNA synthesised from random primers, followed by end repair, dA-tailing, adaptor ligation, and PCR enrichment. Libraries were sequenced at the Leeds Institute for Molecular Medicine (LIMM) using Illumina 3000 system, using paired-end sequencing to generate ~ 50 million reads per sample. Sequence reads were trimmed to remove any adapter sequences using Cutadapt version 1.8.3 [57] and then aligned to version GRCm38 of the mouse genome using HISAT2 [58]. Transcriptomes were assembled and gene expression quantified using the Tuxedo pipeline (version 2.2.1) [59]. Cufflinks was used to assemble transcriptomes for each sample using the GTF annotation file for the GRCm38 mouse genome (*C57BL/6*), followed by Cuffmerge to merge individual sample transcriptomes. Quantification, normalisation, and differential expression was carried out using Cuffquant, Cuffnorm, and Cuffdiff, respectively. False discovery rate (FDR) was controlled using the Benjamini–Hochberg method in the StatsModels library (v.0.8.0), to generate *q*-values. Genes are stated to differ in expression level when the log_2_(fold change) between two states is either above 1 or below − 1 and FDR *q* < 0.05. Thresholds are illustrated in the volcano plots in Additional file 1: Fig. S2. Volcano plots were generated in Excel. Heat maps were generated using Spyder (v.4.1.4), accessed via Anaconda Navigator (v.1.9.12), using the pandas, seaborn, and matplotlib modules. Transcription units which did not have a numerical value for log_2_(fold change), due to mean expression of 0 in one condition, were manually removed before generating the plots. Individual transcripts per million (TPM) were extracted for biological replicates (independent PEF lines) enabling calculation of means and SEM and comparison between genotypes. Gene Set Enrichment Analysis [33] was performed in Python 3.6 using one-sided Fisher’s exact tests as part of the SciPy library (v.0.19.0).

### Supplementary Information

**Additional file 1: Figure S1.** Comparison of the human and mouse quiescence program. **Figure S2.** Whole genome expression during entry to quiescence. **Figure S3.** Expression changes for the 33 I-DN DREAM complex target genes during quiescence entry. **Figure S4.** H4K20me1 loss in male CIZ1-null cells and features of colony cells.

**Additional file 2: SDataset 1.** Excel file showing mean data with significance indicators for all transcription units that are changed upon entry to quiescence in WT PEFs. Sheet 1, effect of serum withdrawal. Sheet 2, effect of contact inhibition. Sheet 3, core murine quiescence genes. Sheet 5 and 6, GSEA output for up and down regulated genes respectively, showing significance indicators for GO terms used to create the graph shown in Fig 2.

**Additional file 3: SDataset 2.** Excel file showing mean data with significance indicators for all transcription units that are changed upon entry to quiescence in CIZ1-null PEFs. Sheet 1, effect of serum withdrawal. Sheet 2, effect of contact inhibition. Sheet 3, core CIZ1-null murine quiescence genes. Sheet 5 and 6, GSEA output for up and down regulated genes respectively, showing significance indicators for GO terms used to create the graph shown in Fig 2.

**Additional file 4: SDataset 3.** Excel file showing change with significance indicators for all transcription units between a cycling and quiescent state. Sheet 1 and 2, transcription units that are common to both WT and CIZ1-null cells following the effect of serum withdrawal or contact inhibition, respectively. Sheets 3-10, transcription units that change upon entry to quiescence for one genotype but not the other, creating lists for both the serum withdrawal and contact inhibition methods individually with genes split into four categories, those that fail to go up or down (only changed in WT) or those that are inappropriately changed (only changed in CIZ1-null. Sheet 11, core gene set that fail to go up in CIZ1-null PEFs. Sheet 12, core gene set that fail to go down to in CIZ1-null PEFs. Sheet 13, core gene set that are inappropriately upregulated in CIZ1-null PEFs. Sheet 14, core gene set that are inappropriately downregulated in CIZ1-null PEFs.

**Additional file 5: SDataset 4.** Excel file showing GSEA output for the four core gene sets from SDataset 3. Significance indicators for overlap with curated gene sets used to create the graph shown in Fig 2 are indicated. Sheet 1, curated gene set output for core genes that fail to go up in CIZ1-null PEFs. Sheet 2, curated gene set output for core genes that fail to go down in CIZ1-null PEFs. Sheet 3, curated gene set output for core genes that are inappropriately upregulated in CIZ1-null PEFs. Sheet 4, curated gene set output for core genes that are inappropriately down regulated in CIZ1-null PEFs.

**Additional file 6: SDataset 5.** Excel file showing mean data and the size of change with significance indicators for all transcription units from the curated gene set “M149, Fischer DREAM targets” that are inappropriately downregulated in CIZ1-null PEFs during entry to quiescence by both methods (serum withdrawal and contact inhibition). The three transcription units relating to gene ids; XLOC_007113, XLOC_012670 and XLOC_028106 were removed from analysis due to other gene names mapping to the same locus. This creates the list of 33 genes. Sheet 2, GSEA output for the 33 IDN dream target genes in Sheet 1 and their overlap with GO terms. Additional sheets show the same data for the second (Lee BMP2 targets DN), third (Marson bound by E2F4 unstimulated), fourth (Gobert oligodendrocyte differentiation UP) and fifth (Zhang TLX targets 60hr DN) most significant overlapping gene sets (related to Fig. 2H).

**Additional file 7: Table S1.** Core set of 33 DREAM complex target genes that are inappropriately downregulated during quiescence entry in CIZ1-null cells. **Table S2.** Primary cell populations. **Table S3.** Antibodies.

**Additional file 8.** Original, unedited western blots for Fig. 3F, 4D and Fig. S4A.

## Data Availability

Transcriptome data is available at the SRA repository under the BioProject PRJNA862366 [60]. All other relevant data supporting the key findings of this study are available within the article and its supplementary information files or from the corresponding author upon reasonable request.

## Abbreviations

CIZ1: Cip1-interacting zinc finger protein 1
DREAM: Dimerization partner, RB-like, E2F and multi-vulval class B
Xi: Inactive X chromosome
lncRNA: Long non-coding RNA
PTMs: Post-translational modifications
PEFs: Primary embryonic fibroblasts
WT: Wild-type
SW: Serum withdrawal
AB: Serum add back
CI: Contact inhibition
mCQG: Murine core quiescence genes
GSEA: Gene set enrichment analysis
GO: Gene ontology
TPM: Transcripts per million
F-UP: Fail to go up
F-DN: Fail to go down
I-UP: Inappropriately upregulated
I-DN: Inappropriately downregulated
H4K20me1: Mono-methylation of histone H4 at lysine 20
H3K27me3: Tri-methylation of histone H3 at lysine 27
H2AK119Ub1: Ubiquitylation of histone H2A at lysine 119
TTFs: Dermal fibroblasts from tail tips
pATM: Phosphorylation of ataxia telangiectasia mutated
γH2AX: Phosphorylated histone H2AX
pCHK1: Phosphorylated checkpoint kinase 1
dox: Doxycycline
