# Supplementary material for: Epigenetic instability caused by absence of CIZ1 drives transformation during quiescence cycles
Source: BMC Biol. 2023 Aug 15;21:175. doi: 10.1186/s12915-023-01671-6 (PMC10426085; doi:10.1186/s12915-023-01671-6)
Supplement: Supplementary file 1 — Additional file 1: Figure S1. Comparison of the human and mouse quiescence program. Figure S2. Whole genome expression during entry to quiescence. Figure S3. Expression changes for the 33 I-DN DREAM complex target genes during quiescence entry. Figure S4. H4K20me1 loss in male CIZ1-null cells and features of colony cells. [file 12915_2023_1671_MOESM1_ESM.pdf]

Found in mCQG			
Genes (22)			
Timp3	*Ror1*	*Lum*	*Serping1*
Mx2	*Ifitm1*	*Gm2a*	*Fcgrt*
Tgfbr2	*Ctso*	*Ech1*	*Tyms*
Ube2s	*Cenpa*	*Cdkn3*	*Cks2*
Tk1	*Ccnb1*	*Cdc20*	*Tfrc*
Lmnb1	*Has2*		

Found in mCQG under alternative name	
Genes (3)	
Human Q program	Mouse Q program
Garp	*Lrrc32*
Dlg7	*Dlgap5*
Pippin	*Csdc2*

Found in mCQG with other genes mapped to same loci	
Genes (3)	
Human Q program	Mouse Q program
S100a4	*S100a4, (Gm42674, S100a3)*
Foxm1	*Foxm1, (4933413g19rik, Nrip2, Rp23-114e15.17)*
Col3a1	*Col3a1, (Ac125167.1)*

(gene) = unrelated gene mapped to the same locus

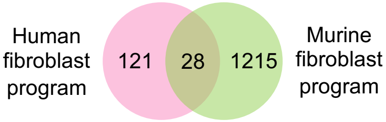

Not found in mCQG but paralogs or genes from the same family present			
Genes (40)			
Human Q	Mouse Q	Human Q	Mouse Q
Glb1	*Glb1l, (Gm27943)*	*Cdh11*	*Cdh19*
Lamc1	*Lamc2*	*Ctnna1*	*Ctnna2*
Fat	*Fat3,4*	*Tp53*	*Trp53inp1*
Fgd1	*Fgd3,4*	*Plcb4*	*Plce1, Plcl1*
Mylk	*Mylk2*	*Itga1*	*Itga5,6*
Il6	*Il11, Il33*	*Il1r1*	*Il1rl1*
Madh1	*Smad7*	*Sept6*	*Sept4, (Gm11492)*
Irp1	*Irp2,6,8*	*Usp14*	*Usp11*
Herc1	*Herc3*	*Adar*	*Adarb1*
Acta2	*Actg2*	*Napg*	*Napb*
Tnfaip1	*Tnfaip8l3*	*Pik3c3*	*Pik3cb*
Itga1	*Itga5,6*	*Nptx1*	*Nptx2*
Bmp1	*Bmp4, Bmp6*	*Plat*	*Plau*
C1r	*C1ra*	*C1s*	*C1s1*
Trim22	*multiple Trim genes*	*Hist1h4c*	*multiple Hist1 genes*
Ccnd2	*multiple Ccn genes*	*Abcd3*	*multiple Abc genes*
Ptprk	*multiple Ptpr genes*	*Col4a5*	*multiple Col genes*
Serpinf1	*multiple Serpin genes*	*Ppp2cb*	*multiple Ppp genes*
Tnfrsf1a	*multiple Tnfrsf genes*	*Ptprg*	*multiple Ptpr genes*
Gpr37	*multiple Gpr genes*	*Plekhc1*	*multiple Plekh genes*

Figure S1 (related to Fig.2). **Comparison of the human and mouse quiescence program.** The gene list described as the murine quiescence program (Additional file 2: SDataset 1) was compared to the gene list described as the human quiescence program [4]. 28 genes were common to both quiescence programs.

A

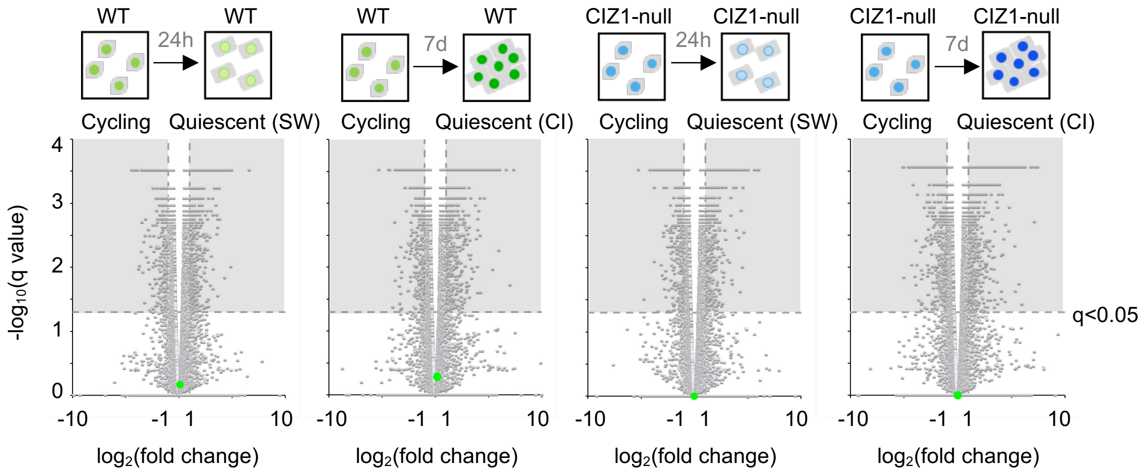

Threshold	Genes affected Total	Up	Down	Genes affected Total	Up	Down	Genes affected Total	Up	Down	Genes affected Total	Up	Down
q < 0.05 $-1>\log_2 fc>1$	1954	1086	868	2072	1218	854	2078	1071	1007	2514	1357	1157

B

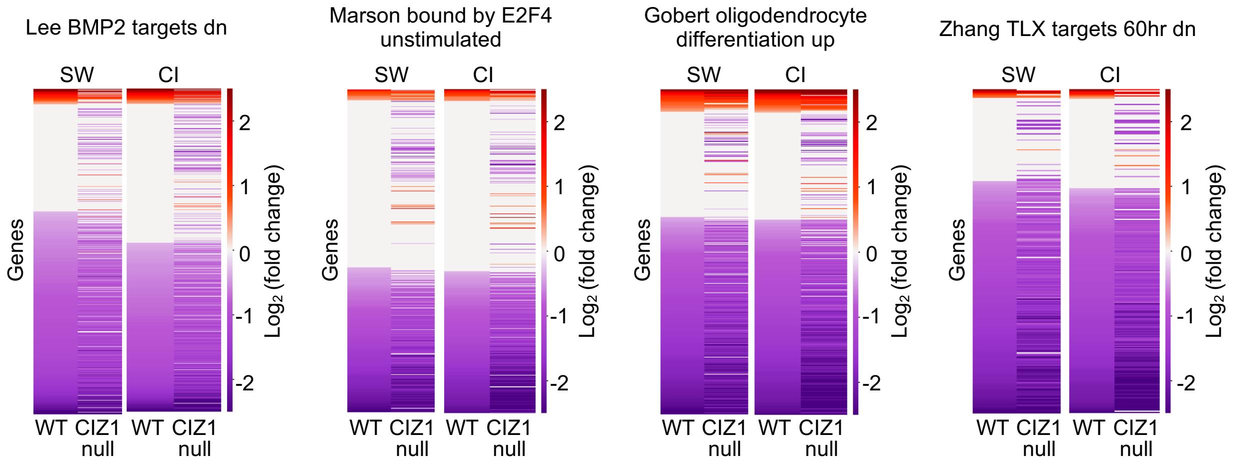

Figure S2 (related to Fig.2). **Whole genome expression during entry to quiescence**.

A) Volcano plots showing the mean fold change in transcript level against significance (q-value) for all 43834 genes in WT and CIZ1-null PEFs over the quiescence transition for both serum withdrawal (SW) and contact inhibition (CI) protocols, as indicated. CIZ1 is highlighted on each plot in green. Below each plot are the number of genes that reach the threshold $-1>\log_2 fc>1$ and the specified significance threshold of $\log_2 fc$ FDR q<0.05. The comparison being made in each plot is indicated in the schematic above. Data is given in Additional file 2: SDataset 1 and Additional file 3: SDataset 2. B) Heat maps compare $\log_2$(fold change) in expression during quiescence entry between WT and CIZ1-null PEFs, for the two quiescent methods, for genes defined in the 4 gene sets; Lee BMP2 targets DN, Marson bound by E2F4 unstimulated, Gobert oligodendrocyte differentiation UP and Zhang TLX targets 60hr DN. Transcription units are ordered by WT $\log_2$(fold change) SW or CI; upregulated (red), downregulated (purple), and where q>0.05 (white).

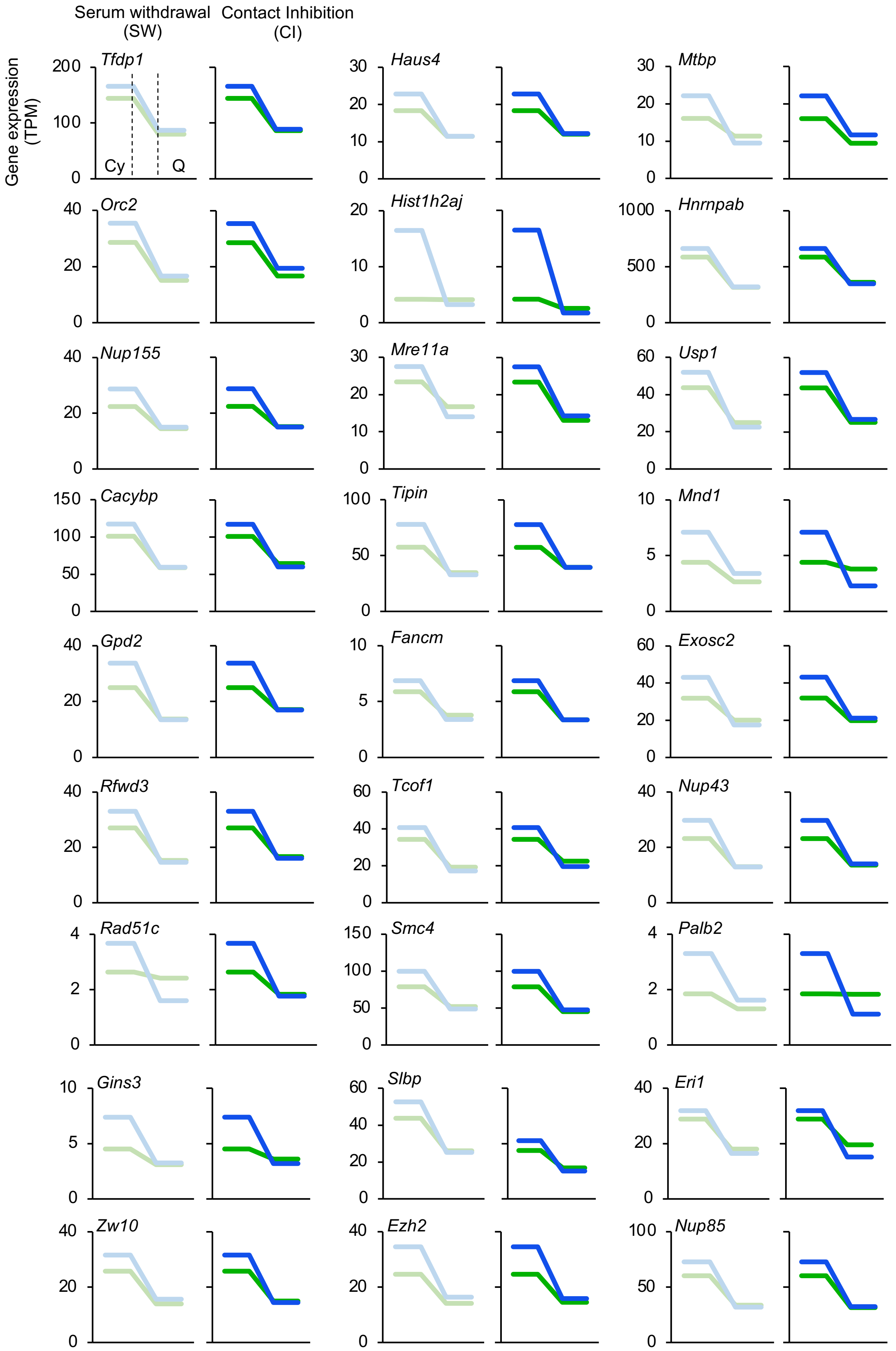

Serum withdrawal (SW)
Contact Inhibition (CI)
Gene expression (TPM)
Cy
Q
Tfdp1
Haus4
Mtbp
Orc2
Hist1h2aj
Hnrnpab
Nup155
Mre11a
Usp1
Cacybp
Tipin
Mnd1
Gpd2
Fancm
Exosc2
Rfwd3
Tcof1
Nup43
Rad51c
Smc4
Palb2
Gins3
Slbp
Eri1
Zw10
Ezh2
Nup85

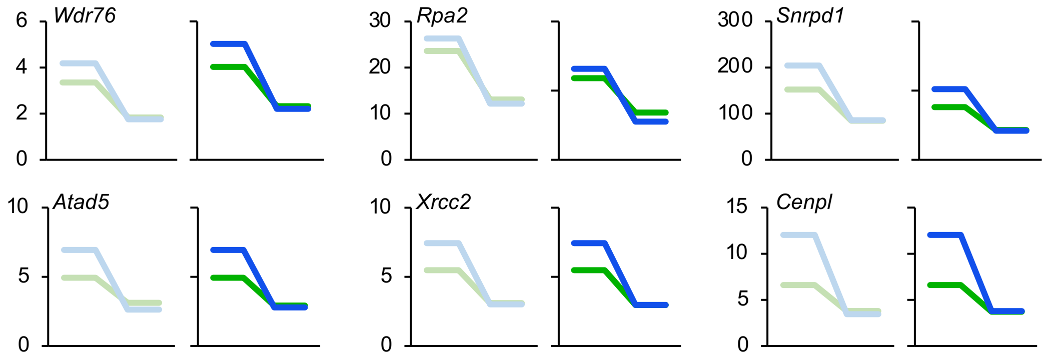

Figure S3 (related to Fig.3). **Expression changes for the 33 I-DN DREAM complex target genes during quiescence entry.** Graphs show mean expression levels (Mean Transcripts Per Million (TPM)) of all 33 I-DN DREAM complex target genes before (cycling, Cy) and after (quiescence, Q) contact inhibition (right) and serum withdrawal (left). All genes see a drop in TPM at the end of both quiescence protocols and in both genotypes, but this fall is only significant for CIZ1-null cells. There is no significant difference between the cycling expression levels for the two genotypes or between the quiescent expression levels, for both protocols and for all genes.

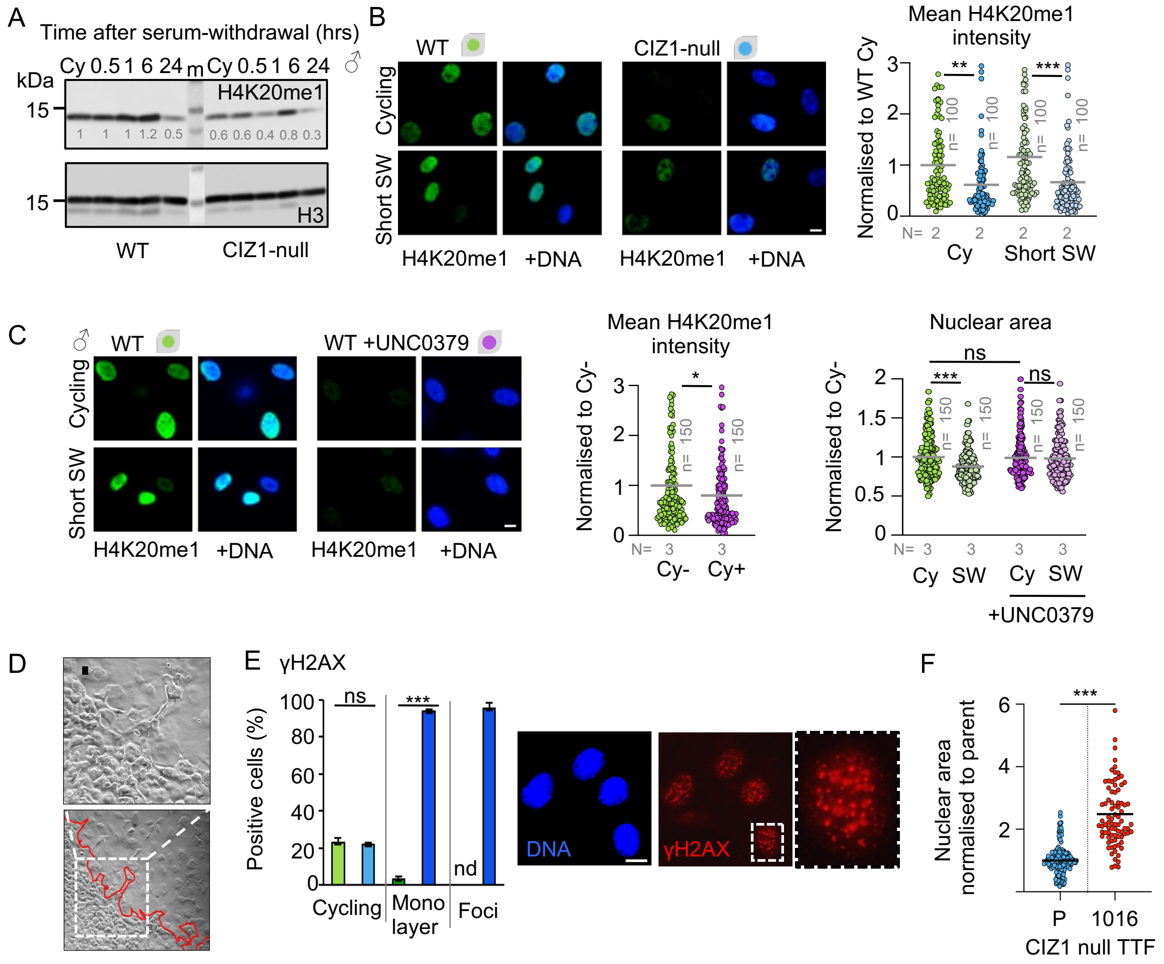

Figure S4 (related to Fig.4 and 5). **H4K20me1 loss in male CIZ1-null cells and features of colony cells. A.** Western blot illustrating H4K20me1 levels over a 24-hour SW time course for WT and CIZ1-null male PEFs, and Histone H3 loading control. Values represent quantification by densitometry where all values have been normalised to Histone H3 and are relative to WT cy (lane 1). **B.** Mean H4K20me1 immunofluorescence signal in nuclei from male WT and CIZ1-null PEFs in a cycling state and following a short SW. **C.** Effect of UNC0379 on mean H4K20me1 immunofluorescence signal and nuclear area before and after a short SW in male WT PEFs. **D.** High magnification bright field image of the edge (red in right image) of a CIZ1-null TTF colony. Scale bar is 20µm. **E.** Cycling, monolayer and foci TTFs from WT and CIZ1-null (n=2) showing proportion of cells stained with γH2AX. Representative images show γH2AX expression (red) in foci in contact inhibited CIZ1-null TTFs at the end of the time course. **F.** Nuclear area quantification of CIZ1-null parent TTFs (P, blue) and a spontaneously immortalised TTF line (1016, red). Results are compared by t-test (C(left),E,F) or two-way ANOVA (B,C(right)) where ns denotes no significant difference, * p<0.05, ** p<0.01, *** p<0.001. DNA is stained with DAPI (blue) and scale bars represent 10µm.
