# Supplementary material for: Epigenetic instability caused by absence of CIZ1 drives transformation during quiescence cycles
Source: BMC Biol. 2023 Aug 15;21:175. doi: 10.1186/s12915-023-01671-6 (PMC10426085; doi:10.1186/s12915-023-01671-6)
Supplement: Supplementary file 7 — Additional file 7: Table S1. Core set of 33 DREAM complex target genes that are inappropriately downregulated during quiescence entry in CIZ1-null cells. Table S2. Primary cell populations. Table S3. Antibodies. [file 12915_2023_1671_MOESM7_ESM.pdf]

Additional file 7.

Table S1 (related to Fig.3) Core set of 33 DREAM complex target genes that are inappropriately downregulated during quiescence entry in CIZ1-null cells.

33 DREAM target genes I-DN	UniProtKB identifier	GO-biological process annotations	
Tfdp1: Transcription factor Dp-1	Q08639	15	Cell cycle, regulation of transcription
Haus4: HAUS Augmin like complex subunit 4	Q8BFT2	10	Cell division, centrosome cycle, spindle assembly
Mtbp: MDM2 binding protein	Q8BJS8	9	Cell cycle, regulation of protein ubiquitination, protein localisation to kinetochore
Orc2: Origin recognition complex subunit 2	Q60862	2	DNA replication
Hnrnpab: Heterogeneous nuclear ribonucleoprotein A/B	Q99020	9	Regulation of transcription, regulation of gene expression, mRNA modification
Nup155: Nucleoporin 115	Q99P88	14	mRNA and protein transport, nuclear envelope organisation
Mre11a: Meiotic recombination 11 homolog A	Q61216	49	Double-strand break repair, DNA repair, telomere maintenance, DNA damage checkpoint signalling, cellular response to DNA damage stimulus
Usp1: Ubiquitin carboxyl-terminal hydrolase 1	Q8BJQ2	20	DNA repair, protein deubiquitination, cellular response to DNA damage stimulus
Cacybp: Calcyclin-binding protein	Q9CXW3	6	Negative regulation of cell death, positive regulation of DNA replication
Tipin: TIMELESS-interacting protein	Q91WA1	23	DNA replication checkpoint signalling, replication fork protection/arrest, cell cycle phase transition, positive regulation of cell population proliferation
Gpd2: Glycerol-3-phosphate dehydrogenase	Q64521	8	Glycerol-3-phosphate metabolic process, NADH metabolic process, gluconeogenesis
Fancm: Fanconi anemia group M protein homolog	Q8BGE5	20	Cellular response to DNA damage stimulus, DNA repair, replication fork processing, positive regulation of protein monoubiquitination
Exosc2: Exosome Component 2	Q8VBV3	26	RNA processing, positive regulation of cell growth
Rfwd3: E3 ubiquitin-protein ligase	Q8CIK8	31	Double-strand break repair via HR, protein ubiquitination, mitotic G1 DNA damage checkpoint signalling, replication fork processing, chromosome breakage
Tcof1: Treacle protein	O08784	8	Neural crest cell development/formation, regulation of translation
Nup43: Nucleoporin 43	P59235	7	Protein transport, cell division, mRNA transport, chromosome segregation, cell cycle
Rad51c: RAD51 Paralog C	Q924H5	22	Double-strand break repair via HR, DNA repair, DNA recombination, positive regulation of G2/M transition
Smc4: Structural maintenance of chromosomes protein 4	Q8CG47	20	Chromosome condensation, cell division, cell cycle, single strand break repair, kinetochore organisation, chromosome segregation

Slbp: Histone RNA hairpin-binding protein	P97440	10	mRNA processing/transport, cap-dependent translational initiation
Eri1: 3'-5' exoribonuclease 1	Q7TMF2	5	Histone mRNA catabolic process, rRNA processing, gene silencing by RNA
Zw10: Centromere/kinetochore protein zw10 homolog	O54692	30	Mitotic sister chromatid segregation, regulation of exit from mitosis, mitotic spindle assembly, checkpoint signalling, protein transport, cell division
Ezh2: Enhancer of zeste homolog 2	Q61188	92	Histone H3-K27 methylation, negative regulation of transcription/gene expression, chromatin organisation, positive regulation of MAP kinase activity
Nup85: Nucleoporin 85	Q8R480	14	Protein import into nucleus, mRNA export from nucleus, positive regulation of transcription
Wdr76: WD repeat-containing protein 76	A6PWY4	5	Cellular response to DNA damage stimulus, regulation of DNA damage checkpoint
Gins3: GINS Complex Subunit 3	Q9CY94	12	DNA replication, DNA unwinding involved in DNA replication
Rpa2: Replication protein A	Q62193	29	Double-strand break repair via HR, nucleotide-/base-excision repair, mismatch repair, DNA replication, telomere maintenance, regulation of DNA damage checkpoint, protein localisation to chromosome, DNA recombination
Snrpd1: Small Nuclear Ribonucleoprotein D1	P62315	11	Spliceosomal snRNP assembly, mRNA splicing, RNA processing
Atad5: ATPase family AAA domain-containing protein 5	Q4QY64	25	Positive regulation of DNA replication and cell cycle G2/M phase transition, cellular response to DNA damage stimulus, intrinsic apoptotic signalling pathway in response to DNA damage
Xrcc2: X-Ray repair cross complementing 2	Q9CX47	32	Double-strand break repair via HR, strand invasion, cell cycle, DNA repair, cellular response to DNA damage stimulus, DNA recombination
Palb2: Partner And Localiser Of BRCA2	Q3U0P1	17	Double-strand break repair via HR, cellular response to DNA damage stimulus, DNA recombination, DNA repair
Mnd1: Meiotic Nuclear Divisions 1	Q8K396	4	DNA recombination, cell cycle
Cenpl: Centromere protein L	Q3U3S3	1	Assembly of kinetochore proteins, mitotic progression, chromosome segregation
Hist1h2aj: Histone H2A type 1-J	Q99878	2	Heterochromatin assembly

Unique UniProtKB identifiers are shown, along with the overarching GO biological process annotation for each identifier, collected using QuickGO (www.ebi.ac.uk/QuickGO).

Table S2: Primary cell populations.

Strain number	Source	Genotype	Sex
101	Tail-tip fibroblast	WT (+/+)	M
126	Tail-tip fibroblast	WT (+/+)	M
133	Tail-tip fibroblast	WT (+/+)	M
1017	Tail-tip fibroblast	CIZ1-null (-/-)	M
1018	Tail-tip fibroblast	CIZ1-null (-/-)	M
1016	Tail-tip fibroblast spontaneously immortalised	CIZ1-null (-/-)	M
13.1	Primary embryonic fibroblast	WT (+/+)	F
13.8	Primary embryonic fibroblast	WT (+/+)	F
14.4	Primary embryonic fibroblast	WT (+/+)	F
45.1fc	Primary embryonic fibroblast	WT (+/+)	F
45.1ff	Primary embryonic fibroblast	WT (+/+)	F
13.49	Primary embryonic fibroblast	WT (+/+)	F
13.31	Primary embryonic fibroblast	WT (+/+)	F
14.2	Primary embryonic fibroblast	CIZ1-null (-/-)	F
13.15	Primary embryonic fibroblast	CIZ1-null (-/-)	F
13.17	Primary embryonic fibroblast	CIZ1-null (-/-)	F
30.3ca	Primary embryonic fibroblast	CIZ1-null (-/-) with inducible CIZ1 transgene	F
41.2fa	Primary embryonic fibroblast	CIZ1-null (-/-) with inducible CIZ1 transgene	F
14.19	Primary embryonic fibroblast	CIZ1-null (-/-)	F
13.42	Primary embryonic fibroblast	CIZ1-null (-/-)	F
13.59	Primary embryonic fibroblast	CIZ1-null (-/-)	F
13.2	Primary embryonic fibroblast	WT (+/+)	M
13.4	Primary embryonic fibroblast	WT (+/+)	M
45.1fb	Primary embryonic fibroblast	WT (+/+)	M
13.20	Primary embryonic fibroblast	WT (+/+)	M
13.30	Primary embryonic fibroblast	WT (+/+)	M
13.6	Primary embryonic fibroblast	CIZ1-null (-/-)	M
16.5de	Primary embryonic fibroblast	CIZ1-null (-/-)	M
13.45	Primary embryonic fibroblast	CIZ1-null (-/-)	M
13.38	Primary embryonic fibroblast	CIZ1-null (-/-)	M

Table S3: Antibodies.

Antibody	Concentration		Source
	IF	Western	
Lamin B2	1:100	-	Invitrogen, 33-2100
SMC2	-	1:1000	Biorbyt, orb542536
SMC4		1:1000	Novus Biologicals, NBP1-86635
Histone H3	-	1:10000	Abcam, ab1791
H4K20me1	1:1000	1:1000	Active motif, 39728
CIZ1	1:1000	-	1794 [14]
H3K27me3	1:2000	-	Cell signalling technology, C36B11
pATM	1:4000	-	Abcam, 2888
γH2AX	1:2000	-	Upstate, 05-636
pCHK1	1:100	-	Cell signalling technology, 2348S
