# Supplementary material for: Epigenetic instability caused by absence of CIZ1 drives transformation during quiescence cycles
Source: BMC Biol. 2023 Aug 15;21:175. doi: 10.1186/s12915-023-01671-6 (PMC10426085; doi:10.1186/s12915-023-01671-6)
Supplement: Supplementary file 8 — Additional file 8. Original, unedited western blots for Fig. 3F, 4D and Fig. S4A. [file 12915_2023_1671_MOESM8_ESM.pdf]

Additional file 8

Uncropped blots related to Fig.3F

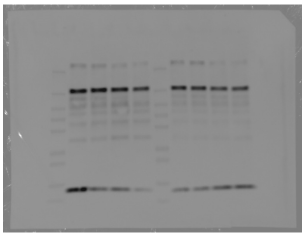

SMC2

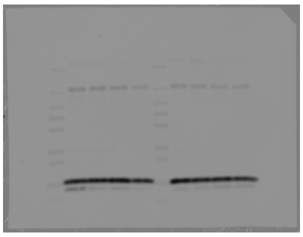

Re-probe Histone H3

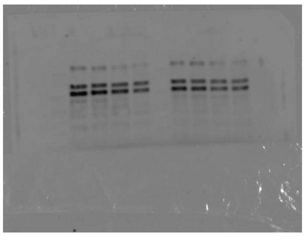

Re-probe SMC4

Uncropped blots related to Fig.4D

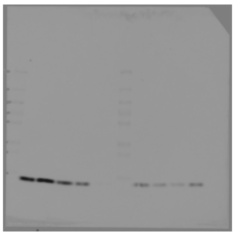

H4K20me1

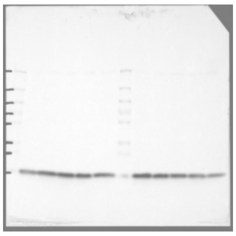

Re-probe: Histone H3

Uncropped blots related to Fig.S4A

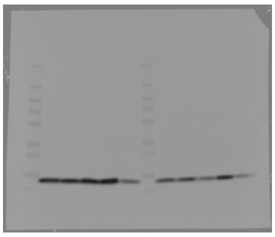

H4K20me1

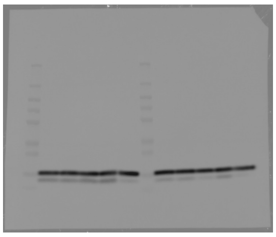

Re-probe: Histone H3
